# Green Synthesis of ZnO Nanoparticles Using Fresh and Dried *Rumex acetosa* Leaf Extracts: Characterization and Antibacterial Activity

**DOI:** 10.3390/nano16211441

**Published:** 2026-11-06

**Authors:** Gül Bayram, Sevda İldan Özmen, Taylan Bozok, Nuran Delialioğlu

**Affiliations:** 1 Department of Medical Services and Techniques, Vocational School of Health Services, Mersin University, 33343 Mersin, Türkiye; 2 Cosmetics Cleaning and Chemical Products Production Education, Application and Research Center, Mersin University, 33343 Mersin, Türkiye; 3 Advanced Technology Education Research and Application Center, Mersin University, 33343 Mersin, Türkiye; sevdaildan@mersin.edu.tr; 4 Department of Chemistry and Chemical Processing Technologies, Technical Science Vocational School, Mersin University, 33343 Mersin, Türkiye; 5 Department of Medical Microbiology, Faculty of Medicine, Mersin University, 33343 Mersin, Türkiye; taylanbozok@hotmail.com (T.B.); nurandelialioglu@mersin.edu.tr (N.D.)

**Keywords:** *Rumex acetosa*, zinc oxide nanoparticles, physicochemical characterization, antibacterial activity

## Abstract

Background: In this study, we aimed to synthesize zinc oxide nanoparticles (ZnO NPs) using aqueous extracts of fresh and dried *Rumex acetosa* leaves and characterize their physicochemical properties and antibacterial activities. Methods: Nanoparticles were characterized using UV–Vis, FE-SEM, EDX, DLS, zeta potential measurements, XRD, and FT-IR spectroscopy. Antibacterial activity was evaluated against five bacterial reference strains using a resazurin microdilution assay to determine minimum inhibitory concentrations (MICs). Results: ZnO NPs from both extracts were spherical and had a hexagonal wurtzite structure. For the fresh and dried leaf preparations, respectively, particle sizes were 12–23 and 19–46 nm, hydrodynamic diameters were approximately 51 and 69 nm, and zeta potentials were approximately −24 and −14 mV. Both samples showed characteristic ZnO UV absorption, band gaps of 3.17 and 3.15 eV, respectively, and Zn–O stretching near 435 cm^−1^. EDX detected carbon in the fresh leaf preparation. Both preparations inhibited all tested bacteria, with MICs ranging from 14.20 to 113.64 µg/mL for fresh leaf and from 7.10 to 113.64 µg/mL for dried leaf preparations. Conclusions: Dried leaf extract-derived ZnO NPs may be evaluated for photocatalysis, UV protection, and sensing, while the antibacterial activity of fresh leaf extract-derived ZnO NPs suggests potential for coatings, wound dressings, and food packaging.

## 1. Introduction

Rapid advancements in nanotechnology have enabled the development of nanoparticles whose size, shape, and surface properties can be controlled, creating a wide range of applications from biomedical to industrial processes [1]. Nanoparticles exhibit unique morphological, optical, catalytic, and biological functions that differ from bulk material due to their high surface area/volume ratios and size-dependent physicochemical properties [2], characteristics that allow nanoparticles to be used widely in drug delivery systems, biomedical imaging, diagnostic applications, antimicrobial therapies, cancer treatment, electronics, energy, and environmental technologies. However, despite their broad application potential, traditional nanoparticle synthesis methods often use toxic chemicals, raising significant environmental and biological safety concerns. To overcome these limitations and promote environmentally friendly production processes, plant extract-mediated green synthesis is increasingly used as a sustainable nanoparticle production approach [3]. Metal and metal oxide nanoparticles obtained by green synthesis are attracting attention in various applications because of their superior physicochemical and antimicrobial properties [1,3]. In particular, silver nanoparticles (AgNPs) stand out for their broad antimicrobial potential, while zinc oxide nanoparticles (ZnO NPs), a class of metal oxide nanoparticles, are gaining importance because of their biocompatibility, unique physicochemical properties, and versatile application possibilities [1,4]. During nanoparticle synthesis from plant extracts, phenolic compounds, flavonoids, and terpenoids stabilize nanoparticles and reduce metal ions. In this way, green synthesis methods support the production of cost-effective and potentially biocompatible nanoparticles, offering a sustainable alternative that reduces toxic chemicals and the environmental impact of synthesis processes [5,6]. In recent studies, leaves, seeds, fruit peels, and other plant-derived waste materials have been used to synthesize ZnO nanoparticles more sustainably. Researchers have used extracts of *Punica granatum*, *Alnus nepalensis*, *Clitoria ternatea*, *Camellia sinensis*, and *Elettaria cardamomum* and evaluated their antimicrobial, antioxidant, photocatalytic, and biomedical properties [7,8,9,10,11]. In green synthesis studies, plant material is used either fresh or after drying, depending on availability and storage requirements. However, drying can substantially alter the content and composition of phenolic compounds and other secondary metabolites in leaves [12], and the influence of this pretreatment on the properties of the resulting nanoparticles has rarely been examined systematically within a single plant species [13]. *Rumex acetosa* L. (common sorrel, Polygonaceae) is a widely distributed edible and medicinal plant whose leaves are rich in flavonoids, proanthocyanidins, phenolic acids, and organic acids [14,15]. These compounds contain hydroxyl, carbonyl, and carboxyl groups capable of coordinating Zn^2+^ ions and adsorbing onto growing particles, making *R. acetosa* a promising source of chelating and capping agents for the green synthesis of ZnO nanoparticles. The formation of ZnO NPs proceeds through a complexation–hydrolysis–condensation (precipitation) pathway followed by thermal dehydration. First, Zn^2+^ ions are coordinated by the phenolic and carboxylate groups of the plant extract (Equation (1)), which control the release of free Zn^2+^ and limit uncontrolled growth. In the alkaline medium, Zn^2+^ then reacts with OH^−^ ions to precipitate zinc hydroxide (Equation (2)), which partially dissolves in excess hydroxide to form the soluble zincate complex [Zn(OH)_4_]^2−^ (Equation (3)). In the final step, Zn(OH)_2_ and zincate species undergo condensation and dehydration, particularly during annealing, yielding crystalline ZnO (Equation (4)) [16].

Zn^2+^ + n L ⇌ [Zn(L)_n_]^2+^ (L = phenolic/carboxylate ligands of the extract) (1)

Zn^2+^ + 2 OH^−^ → Zn(OH)_2_ (2)

Zn(OH)_2_ + 2 OH^−^ ⇌ [Zn(OH)_4_]^2−^ (3)

Zn(OH)_2_/[Zn(OH)_4_]^2−^ → ZnO + H_2_O (+ 2OH^−^) (4)

Species of the genus Rumex have been used to synthesize silver nanoparticles with antibacterial and antibiofilm activity [17,18], and more recently, an ethanolic extract of dried Rumex nervosus leaves was used to synthesize ZnO nanoparticles with antibacterial activity [19]. However, to the best of our knowledge, aqueous extracts of *R. acetosa* leaves have not yet been used for the synthesis of ZnO nanoparticles, and the effect of using fresh versus dried Rumex leaves on the properties of the resulting nanoparticles has not been investigated. In this study, we aimed to synthesize ZnO nanoparticles using a green synthesis method with aqueous extracts of fresh and dried *Rumex acetosa* leaves, characterize their physicochemical properties, and evaluate their antibacterial activity.

## 2. Materials and Methods

### 2.1. ZnO NP Synthesis and Characterization

*Rumex acetosa* leaves were purchased from a local market, where it was stated that they had been produced in Antalya, Türkiye, on 8 December 2025. The fresh leaves were thoroughly washed with tap water, rinsed twice with distilled water, and dried at room temperature to remove surface moisture. One portion of the fresh leaves was cut into small pieces and used immediately to prepare the fresh leaf extract. The remaining fresh leaves were dried in an oven at 30 °C for 48 h, ground into a powder, and used for the preparation of the dried leaf extract. The filtered aqueous extracts were used for the subsequent synthesis of ZnO NPs. To prepare the plant extract, 2 g of dried Rumex spp. leaf powder and 10 g of fresh leaves were separately mixed with 100 mL of ultrapure water and heated at 60 °C for 3 h under continuous stirring using a magnetic stirrer. The different masses were chosen to compensate for the water content of the fresh leaves. After cooling to room temperature, the mixtures were filtered through coarse filter paper, and the filtrates were refrigerated (at 4 °C in the dark for 20 h) for use in green synthesis.

Zinc nitrate hexahydrate (Zn(NO_3_)_2_·6H_2_O; 98%, Sigma-Aldrich, St. Louis, MO, USA), sodium hydroxide (NaOH; ≥99, Riedel-de Haën, Germany), and ultrapure water (18.2 MΩ·cm at 25 °C; Milli-Q Reference, Merck Millipore) were used for the synthesis. For the green synthesis of ZnO NPs, 100 mL of 0.1 M zinc nitrate hexahydrate (Zn(NO_3_)_2_.6H_2_O) was prepared and heated to 60 °C. The 100 mL extract obtained from the dry leaves was added dropwise into the zinc nitrate solution under constant stirring, during which the pH of the reaction mixture was maintained between 8 and 10 by adding 0.1 M NaOH solution. After complete addition of the extract, the mixture was heated at 60 °C for 1 h and then left to stand at room temperature for 12 h. The obtained precipitate was purified by centrifugation and washed three times with ultrapure water to remove impurities. The resulting ZnO NP powders were dried in an oven at 60 °C for 12 h, after which the samples were annealed in a muffle furnace at 300 °C for 2 h in air. The same synthesis procedure was applied using the same volume of the fresh leaf extract. 

The ZnO NP powders were dispersed in ultrapure water at 0.1 mg/mL by ultrasonication for 5 min, and absorbance spectra were recorded in the 300–600 nm range using a Thermo Scientific Multiskan GO microplate spectrophotometer (Thermo Fisher Scientific, Waltham, MA, USA), with ultrapure water as the blank. The morphology of the ZnO NPs was examined using a Zeiss Supra 55 field emission scanning electron microscope (FE-SEM; Carl Zeiss, Oberkochen, Germany). The particle size distributions of the ZnO NPs were determined from the FE-SEM images using the ImageJ 1.54d software by measuring the diameters of 100 particles with clearly distinguishable boundaries for each sample. The elemental composition was determined with the energy-dispersive X-ray spectroscopy (EDX) detector attached to the microscope. Prior to analysis, the ZnO NP powders were dispersed onto conductive carbon tapes mounted on aluminum stubs and then sputter-coated with Pt to prevent charging. The hydrodynamic size distribution and zeta potential were determined by dynamic light scattering (DLS) and electrophoretic light scattering, respectively, using a Malvern Zetasizer Nano ZS (Malvern Panalytical, Malvern, UK). The ZnO NP powders were dispersed in ultrapure water at 0.1 mg/mL by ultrasonication for 5 min, and the measurements were performed at 37 °C. The pH values of the suspensions were 6.65 for dried leaf extract-derived ZnO NPs and 4.77 for fresh leaf extract-derived ZnO NPs, and the conductivities were 8.3 µS/cm and 11.8 µS/cm, respectively. The structural properties of the ZnO NP powders were investigated using a Rigaku Smart Lab powder X-ray diffractometer (XRD) (Rigaku, Tokyo, Japan) with CuKα radiation (λ = 1.5406 Å), and a Perkin Elmer UATR Two Fourier-transform infrared (FT-IR) spectrophotometer (PerkinElmer, Waltham, MA, USA).

### 2.2. Antimicrobial Activity

In this study, we evaluated the antibacterial activities of ZnO nanoparticles produced by green synthesis using *Rumex acetosa* extract. American Type Culture Collection (ATCC) reference strains were used to determine the minimum inhibitory concentrations (MICs) of these nanoparticles. The Gram-positive bacterial reference strains used in this study were *Staphylococcus aureus ATCC 29213* and *Enterococcus faecalis ATCC 29212;* the Gram-negative bacterial reference strains were *Escherichia coli ATCC 25922*, *Klebsiella pneumoniae ATCC 27736*, and *Pseudomonas aeruginosa ATCC 27853*. Since ATCC reference strains were used, ethical committee approval was not required.

The modified series microdilution method was applied to evaluate the antimicrobial activities of the tested nanoparticles [20]. Lyophilized bacterial strains were subcultured on Mueller–Hinton Agar, for 18–24 h at 37 °C. The stock solution of ZnO NPs was prepared in DMSO/H_2_O (50%) at 1000 µg/mL. Suspensions of bacterial strains were prepared in sterile physiological saline solution, and their turbidity was adjusted to the 0.5 McFarland standard, corresponding to an absorbance value of 0.08–0.13 at 625 nm. For antibacterial testing, 100 µL of Mueller–Hinton broth (MHB) was added to each well of the 96-well microplates. 100 µL of the nanoparticle suspension under test was added to the first well, followed by a two-fold serial dilution. The negative control well contained nanoparticle suspension and culture medium but no bacterial inoculum. The assay included an uninoculated nanoparticle control containing Mueller–Hinton broth and ZnO nanoparticles, without bacterial inoculum. Briefly, 100 µL of the NP stock suspension (1000 µg/mL) was added to 100 µL of MHB, yielding an NP concentration of 500 µg/mL. DMSO was used as the solvent in the antibacterial assays. As solvent-only controls were not included, the potential influence of DMSO on the results could not be assessed independently. Figure S1 shows a total of nine wells in each horizontal row: eight test wells followed by one uninoculated NP–resazurin control. Figure S2 shows a total of eight wells in each vertical column: seven test wells followed by one uninoculated NP–resazurin control. The concentration series is arranged from left to right in Figure S1 and from top to bottom in Figure S2. In both figures, the final well contains nanoparticles, Mueller–Hinton broth, and resazurin, without bacterial inoculum. Bacterial suspension was added to all wells except the control well. Plates were incubated at 37 °C for 24 h to determine antibacterial activity, with the test performed in duplicate. At the end of the incubation period, 10 µL of Resazurin (199303, Sigma-Aldrich, Saint Louis, MO, USA; 0.1 mg/mL) was added to each well and then incubated at 37 °C for 4 h. A change in Resazurin from blue to pink indicates bacterial metabolic activity, while a maintained blue color indicates that the active substance inhibits bacterial growth and possesses antibacterial properties [21]. 

## 3. Results

### 3.1. Optical Properties

**Figure 1:**
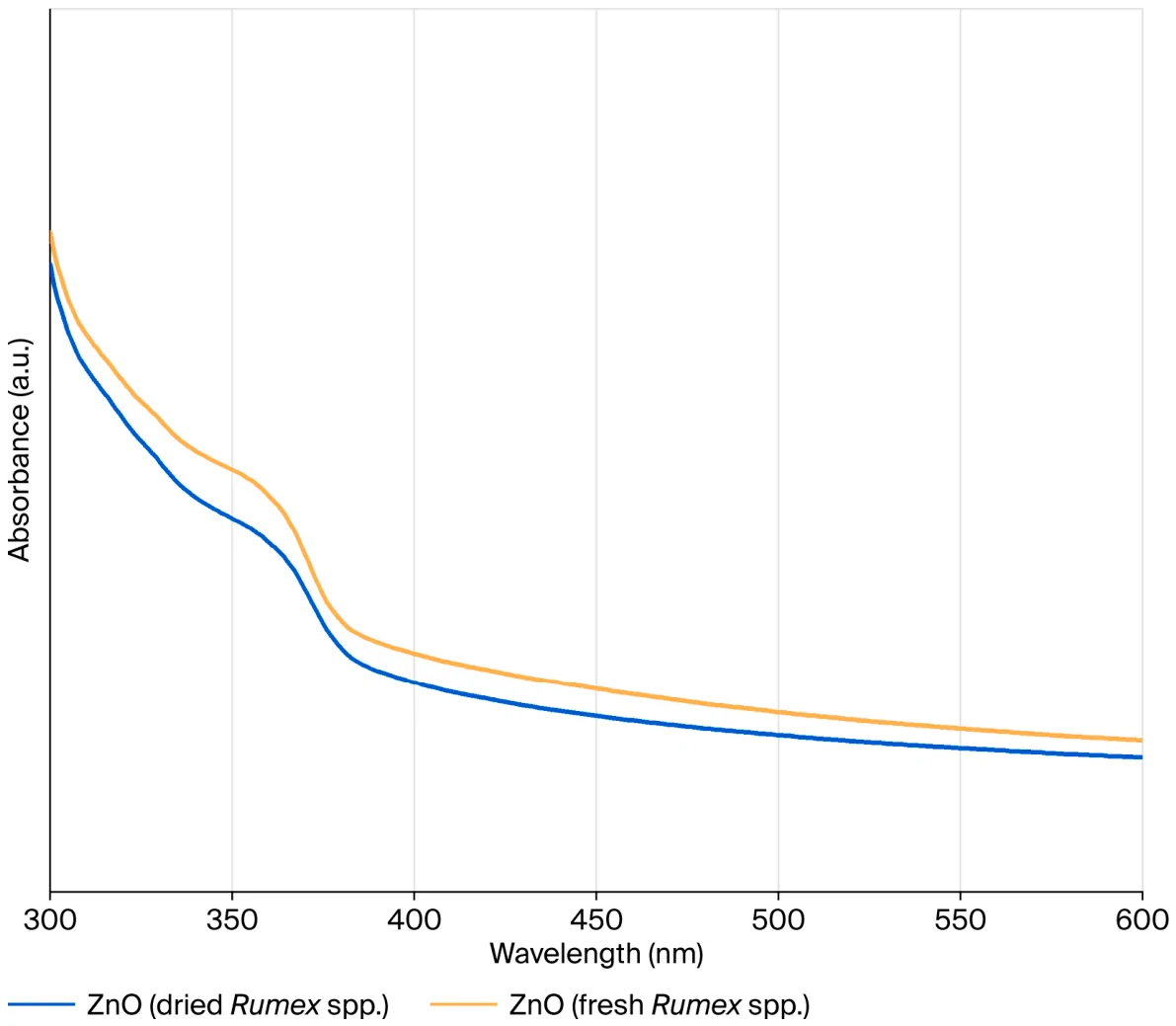
Absorption-wavelength spectra of the ZnO NPs synthesized with dried and fresh *Rumex* spp. extracts.

The optical properties of the ZnO NPs were investigated using absorption spectra in the wavelength range of 300–600 nm. The absorption measurements were carried out with ZnO NP powders dispersed in ultrapure water (Section 2.1) and are shown in Figure 1. The spectra of the ZnO NPs synthesized from dried and fresh *Rumex* spp. showed that both samples exhibited similar broad absorption peaks between 350 and 380 nm, with absorption maxima at 363 nm and 361 nm, respectively. These findings are consistent with those reported in plant-mediated ZnO NP synthesis [22,23,24,25].

**Figure 2:**
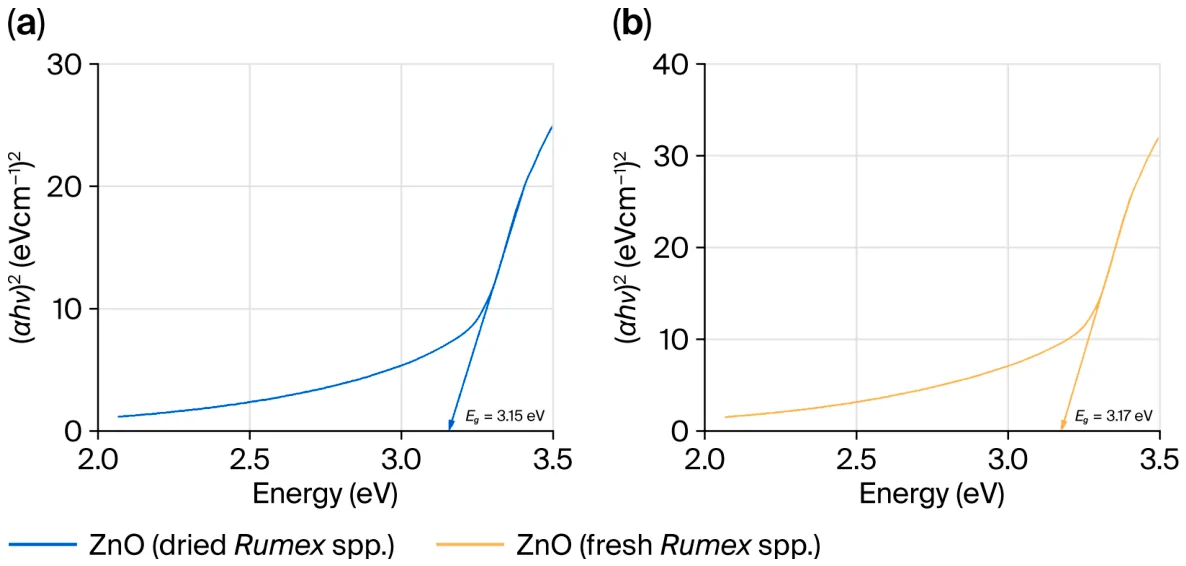
Tauc plots of the ZnO NPs synthesized with (**a**) dried and (**b**) fresh *Rumex* spp. extracts.

The energy band gap (*E_g_*) of the ZnO NPs synthesized with dried and fresh *Rumex* spp. extracts were calculated using the correlation between the absorption coefficient and photon energy [26]:

(5) $$\alpha =A{\left(h\nu -E_{g}\right)}^{n}/h\nu$$

where A is a constant, *α* is the absorption coefficient, *hν* is the photon energy, and the value of *n* is 1/2 because ZnO has a direct optical transition. Figure 2 shows the (*α*hν)^2^-versus-energy (Tauc) plot of the ZnO NPs synthesized with (a) dried and (b) fresh *Rumex* spp. extracts. The *E_g_* value of the ZnO NPs was obtained from the intercept on the energy axis where (*α*hν)^2^ = 0 by extrapolating the linear portion of the plot and was found to be 3.15 eV for ZnO NPs synthesized from dried leaf extract and 3.17 eV for those synthesized from fresh extract. As the results show, there is no significant difference between the *E_g_* values obtained for the two ZnO NPs. These values are lower than those of the bulk ZnO (~3.37 eV), but this indicates that the nanoscale structure and *E_g_* value of the ZnO NPs are consistent with previously reported values for green synthesis [23,25,27,28].

### 3.2. Morphological Properties

**Figure 3:**
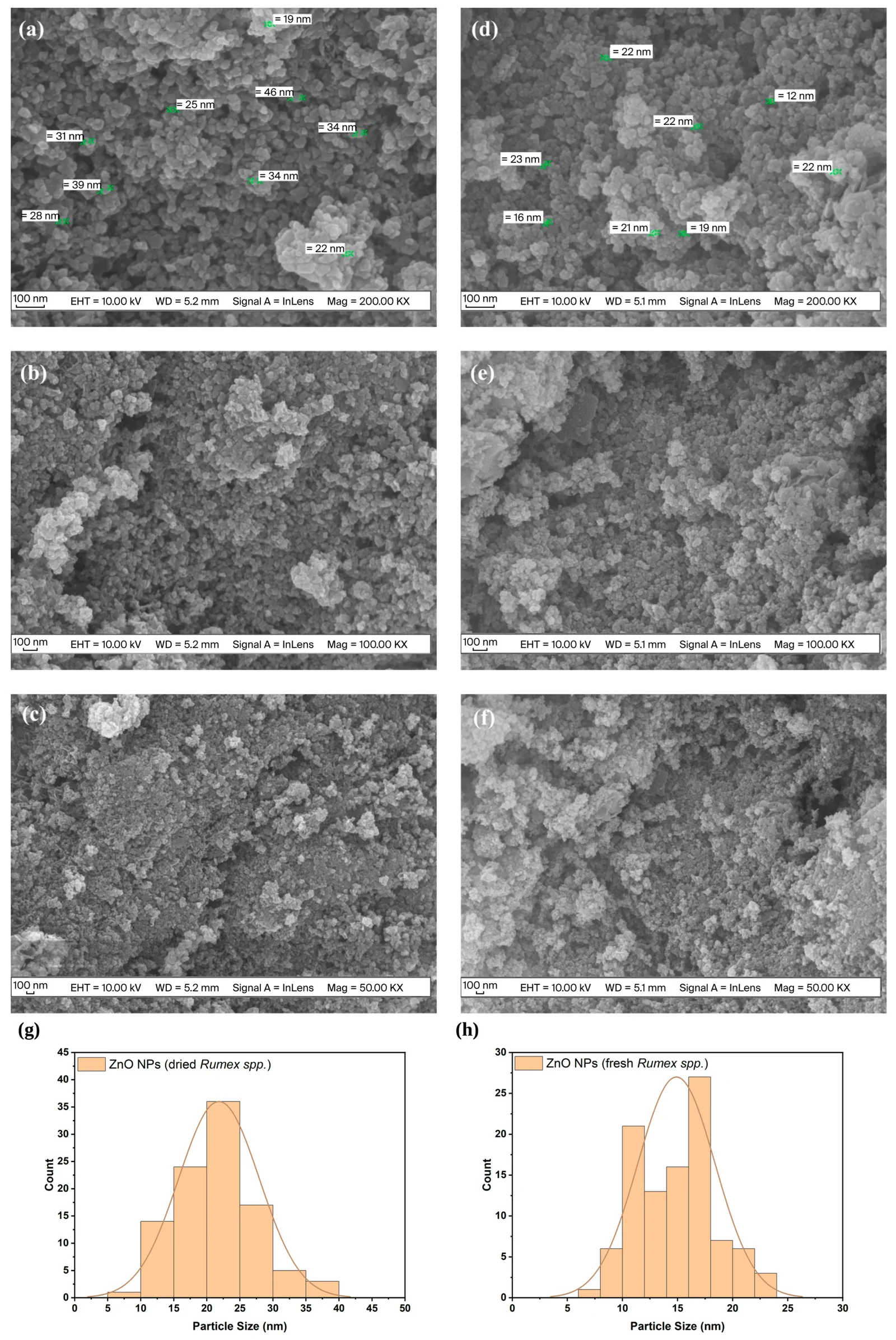
SEM images of the ZnO NPs synthesized with (**a**–**c**) dried and (**d**–**f**) fresh *Rumex* spp. extracts at 200.00 KX, 100.00 KX, and 50.00 KX magnifications, (**g**,**h**) and particle size distribution histograms of the ZnO NPs with Gaussian fits.

The morphological characteristics of the ZnO NPs were investigated by SEM images, with those of the ZnO NPs synthesized with (a–c) dried and (d–f) fresh *Rumex* spp. extracts shown in Figure 3 at 200.00 KX, 100.00 KX, and 50.00 KX magnifications, respectively. The ZnO NPs possess spherical morphologies with nanometer-scale features, as seen in the SEM images. As seen in Figure 3, the primary ZnO particles are not isolated but form larger, irregular agglomerates in both samples. Such agglomeration is typical of ZnO nanopowders and can be attributed to the high surface energy of the nanoparticles, capillary forces during drying of the precipitate, and the formation of interparticle necks during annealing at 300 °C.

The particle size distributions of the ZnO NPs were determined from the FE-SEM images using ImageJ software by measuring the diameters of 100 particles with clearly distinguishable boundaries for each sample. The resulting histograms (Figure 3g,h) show primary particle sizes of 21.6 ± 6.1 nm for the dried leaf extract-derived ZnO NPs and 14.9 ± 3.6 nm for the fresh leaf extract-derived ZnO NPs, demonstrating that the fresh leaf extract produced smaller primary particles with a narrower size distribution. Smaller particle size generally correlates with higher specific surface area, which may improve antimicrobial and anticancer performance.

EDX point analysis was performed to determine the elemental percentages of the ZnO NPs synthesized with dried and fresh *Rumex* spp. Table 1 reports the atomic percentages of the elements. The EDX analysis of Rumex spp. samples demonstrated significant differences in atomic elemental composition between the two ZnO NPs. In the dried leaf extract-derived ZnO NPs zinc (55.68 at.%) and oxygen (44.32 at.%) were the dominant elements, and carbon was not detected; in contrast, the fresh leaf extract-derived ZnO NPs contained carbon (52.71 at.%), oxygen (35.84 at.%), and a substantially lower zinc content (11.45 at.%). The lower-than-expected zinc content may be attributed to residual phytochemicals on the nanoparticle surface, which can mask zinc signals during EDX analysis [23].

### 3.3. Structural Properties

**Figure 4:**
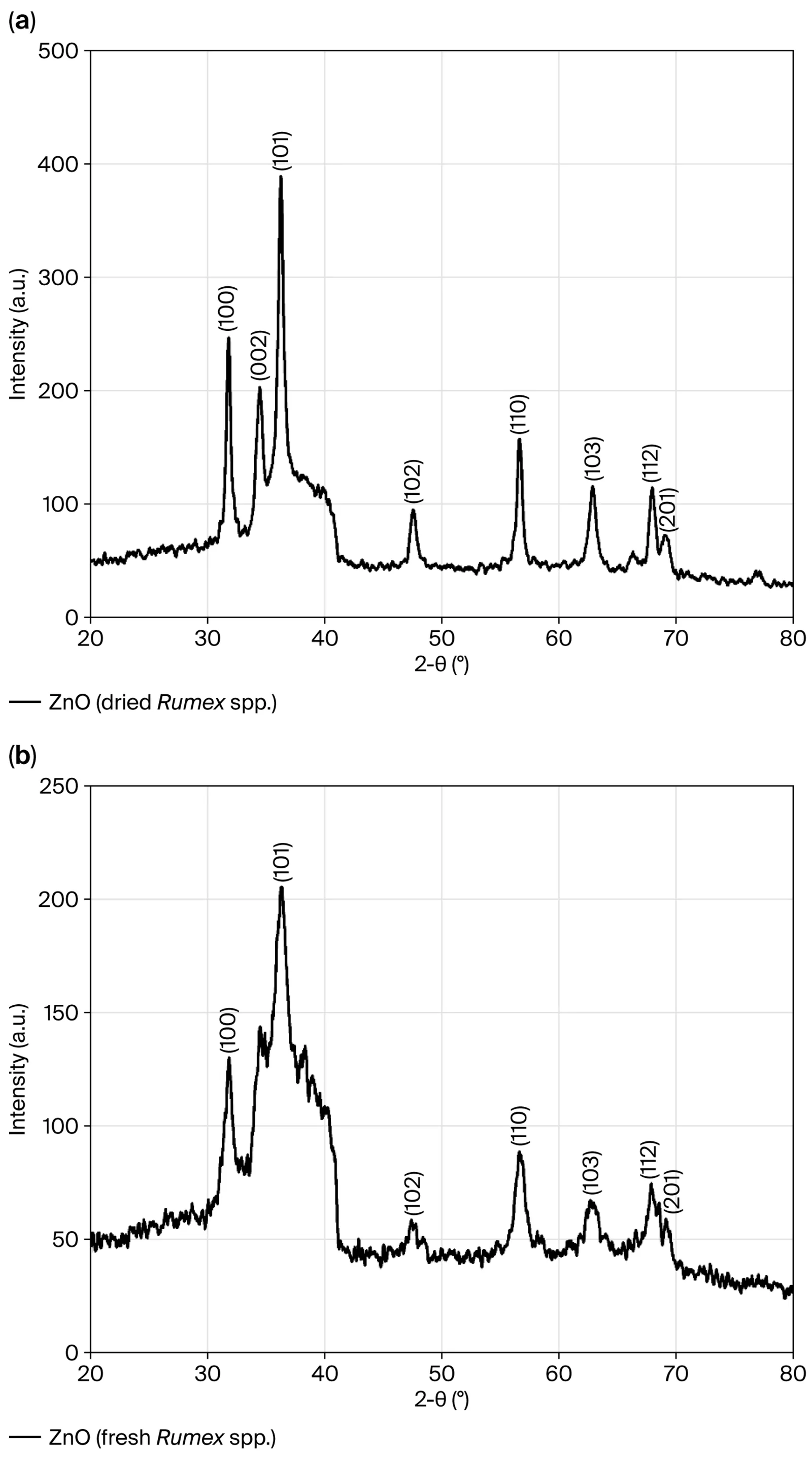
XRD patterns of the ZnO NPs synthesized with (**a**) dried and (**b**) fresh *Rumex* spp. extracts.

The structural properties of the ZnO NPs were examined using X-ray diffraction patterns to determine the ZnO crystal structure. Figure 4 shows the XRD patterns of the ZnO NPs synthesized with (a) dried leaf extract and (b) fresh leaf extract. The diffraction peaks of the ZnO NPs were matched to the crystal structure of ZnO by the JCPDS (Joint Committee on Powder Diffraction Standards) card number 00-005-0664. Their crystal structure was identified as hexagonal wurtzite, and the lattice parameters are given in Figure 4. The stronger, sharper diffraction peaks in dried extract-derived ZnO suggest larger crystallite size and enhanced crystallinity, whereas the broader peaks in fresh extract-derived ZnO indicate smaller nanocrystal dimensions, consistent with SEM and optical analyses. In addition to the wurtzite ZnO reflections, a broad feature appears at 2θ ≈ 37–41° in both ZnO NP diffraction patterns. It is attributed to a small amount of an amorphous or poorly crystalline zinc-containing phase, and its greater intensity in the fresh leaf extract-derived ZnO NPs can be associated with the higher organic content of this sample.

The crystallite size (D), dislocation density (δ), and strain value (ε) of the ZnO NPs were calculated by the following equations:

(6) $$D_{hkl}=\frac{K\lambda }{\beta cos\;\theta }$$

(7) $$\delta =\frac{1}{D_{hkl}^{2}}$$

(8) $$\varepsilon =\frac{\beta cos\;\theta }{4}$$

K is a constant set to 0.9; parameter β represents the Full Width at Half Maximum (FWHM) measured in radians; λ signifies the wavelength of the utilized X-ray (*λ* = 1.54 Å); while θ denotes the Bragg angle [29,30,31,32,33]. The D, δ, and ε values of the ZnO NPs synthesized with dried and fresh *Rumex* spp. leaf extract were calculated using the diffraction peak at approximately 36°, and the results are given in Table 2.

The ZnO NPs crystallite size was calculated to be 16.69 nm and 3.00 nm for dried and fresh *Rumex* spp. extracts, respectively, demonstrating that the use of dried *Rumex* spp. leaf extract promoted the formation of larger, more crystalline ZnO nanoparticles compared to the fresh *Rumex* spp. leaf extract. Furthermore, the lattice parameters and crystallite sizes were also determined using the PDXL software (Rigaku), and the results are presented in Table 3. It should be noted that the crystallite size obtained with PDXL represents the average crystallite size calculated from all indexed diffraction peaks, whereas the Scherrer equation was applied to the most intense (101) reflection. Despite this methodological difference, the crystallite sizes obtained by the two approaches were in good agreement with each other.

**Figure 5:**
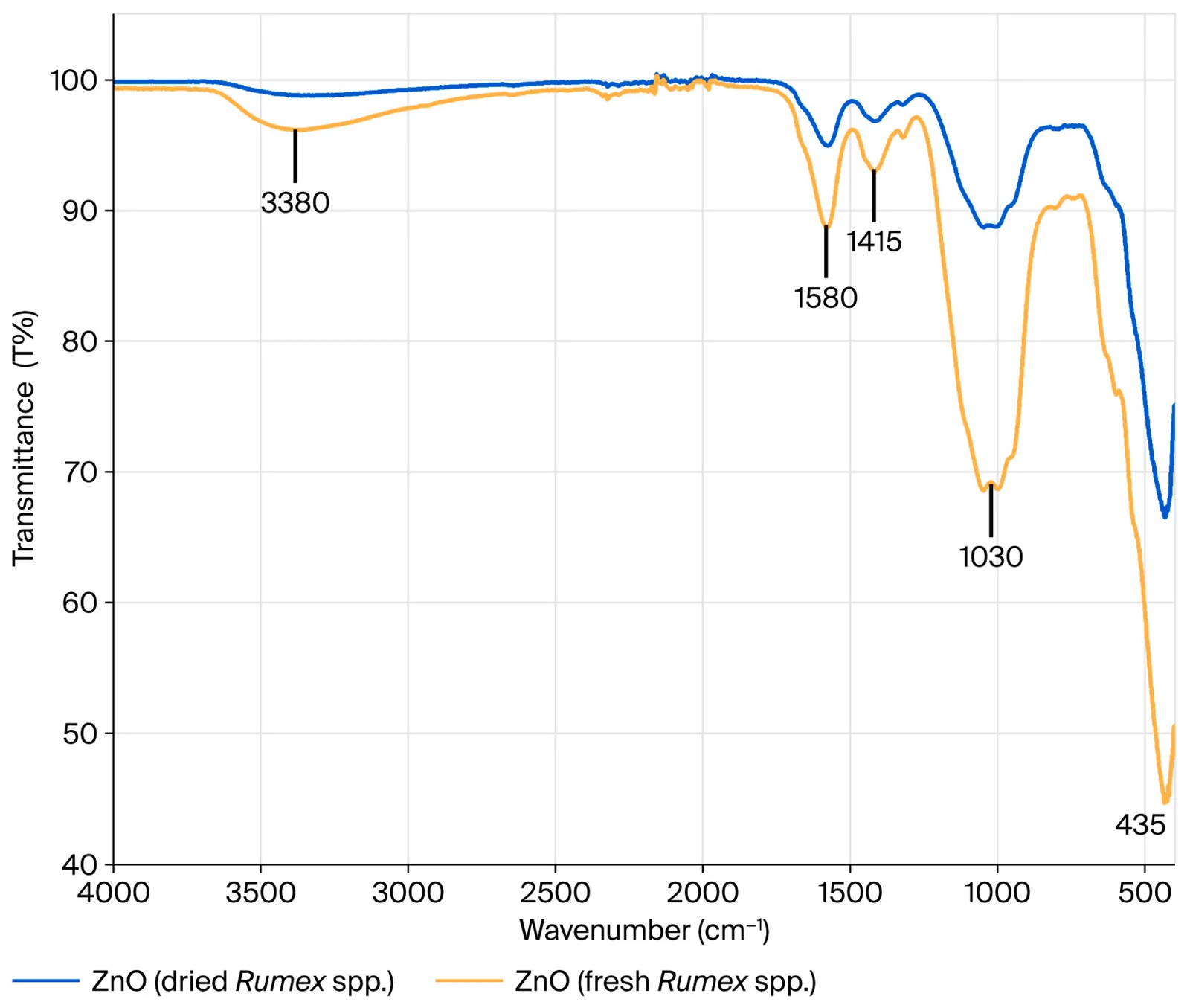
FT-IR transmittance spectra of the ZnO NPs synthesized with dried and fresh *Rumex* spp leaf extract.

FT-IR analysis was used to identify the surface chemistry and interfacial bonding of the ZnO NPs. Figure 5 shows the FT-IR transmittance spectra of the ZnO NPs synthesized with dried and fresh *Rumex* spp. leaf extracts in the range of 4000–400 cm^−1^. The broad peak observed at ~3380 cm^−1^ is attributed to O-H stretching vibrations of hydroxyl groups, while the peaks at ~1580 cm^−1^, 1415 cm^−1^, and 1030 cm^−1^ correspond to the H–O–H bending vibration of adsorbed water, C=O, and C–O stretching vibrations, respectively [23,34]. In the fingerprint region, the peak at ~435 cm^−1^ is attributed to the hexagonal ZnO structure Zn–O stretching vibrations [22,35].

### 3.4. Colloidal Properties

**Figure 6:**
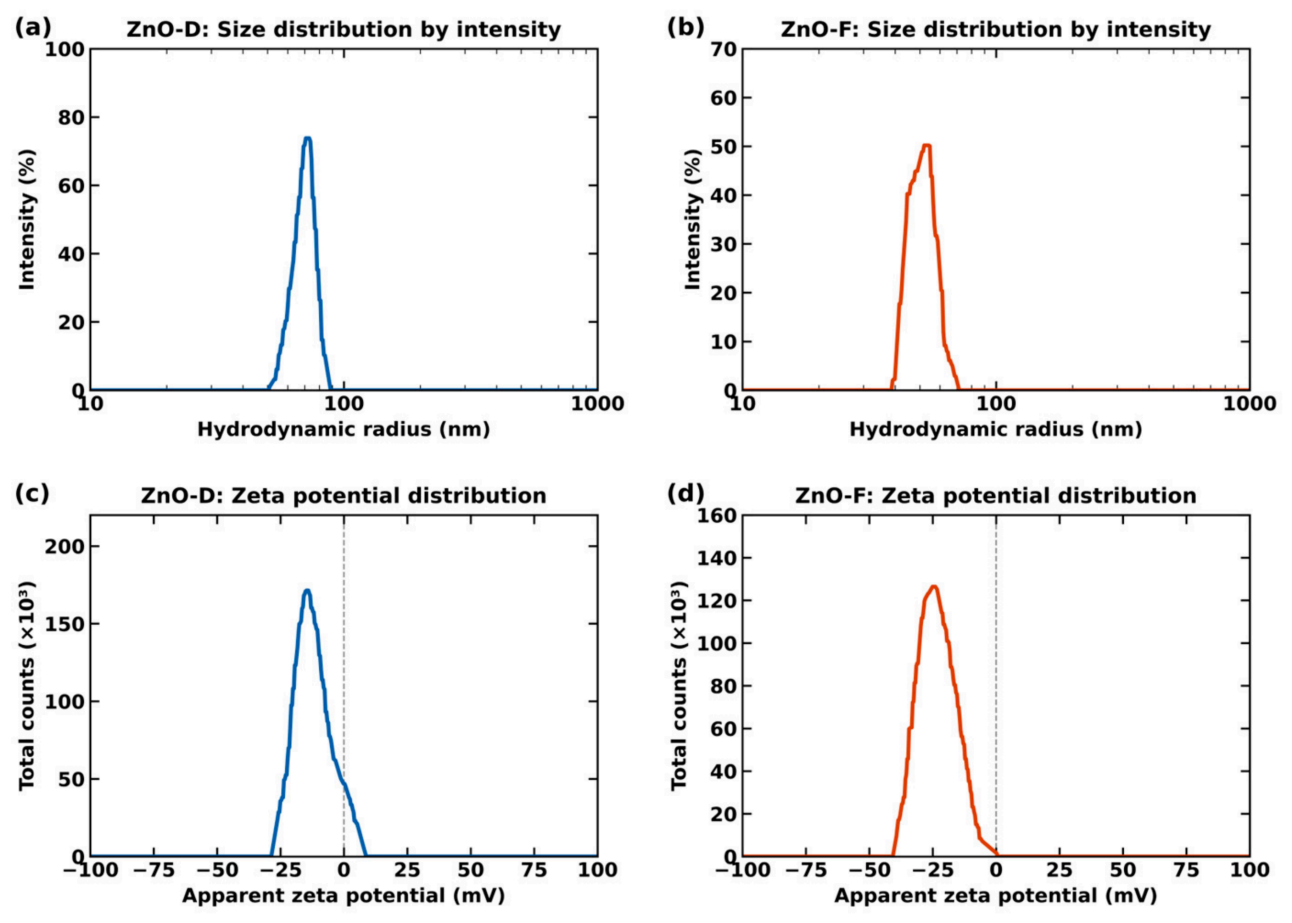
Hydrodynamic size distributions by intensity (**a**) dried leaf extract-derived ZnO NPs and (**b**) fresh leaf extract-derived ZnO NPs, and zeta potential distributions of (**c**) dried leaf extract-derived ZnO NPs and (**d**) fresh leaf extract-derived ZnO NPs, dispersed in water.

The hydrodynamic size distribution peak values of ZnO NPs in aqueous suspension are shown in Figure 6a,b. The ZnO NPs synthesized with dried and fresh *Rumex* spp. leaf extract exhibited a single, narrow population without a contribution from micrometer-sized aggregates. The size distribution peak values were 68 nm (PdI: 0.405) for dried leaf extract-derived ZnO NPs and 51 nm (PdI: 0.566) for fresh leaf extract-derived ZnO NPs, with Z-average radii of 203.80 and 151.04 nm, respectively. These values are markedly larger than the crystallite sizes calculated from XRD and the primary particle sizes observed by FE-SEM, because DLS measures the hydrodynamic diameter of the diffusing species, which includes the solvation layer, adsorbed plant-derived molecules, and small agglomerates of primary particles, whereas XRD reflects the crystallite size and FE-SEM the dimensions of the particles in the dry state [36,37]. Nevertheless, the DLS results follow the same trend as the XRD and FE-SEM data, with the fresh leaf extract-derived ZnO NPs being smaller than the dried leaf extract-derived ZnO NPs.

The zeta potential distributions are shown in Figure 6c,d. Both samples exhibited a single population with negative surface charge, with zeta potentials of approximately −14 mV for the dried leaf extract-derived ZnO NPs and −24 mV for the fresh leaf extract-derived ZnO NPs. Because bare ZnO has an isoelectric point of about pH 9 and would therefore be positively charged at near neutral pH [38], the negative values indicate that the surfaces of both samples are covered by adsorbed anionic species, such as deprotonated carboxyl and phenolic groups of plant-derived compounds, in agreement with the FT-IR results. The more negative surface charge of the fresh leaf extract-derived ZnO NPs can be attributed to the larger amount of organic matter retained on its surface, as indicated by EDX and FT-IR. Since both values are below the commonly used ±30 mV threshold for electrostatically stable dispersions [37], the suspensions show moderate (fresh leaf extract-derived ZnO NPs) to limited (dried leaf extract-derived ZnO NPs) stability and a tendency to agglomerate, consistent with the FE-SEM and DLS results. The stronger electrostatic repulsion, together with possible steric stabilization by the adsorbed biomolecules, may explain the smaller hydrodynamic size of the fresh leaf extract-derived ZnO NPs.

### 3.5. Antibacterial Activity

In this study, the antibacterial activities of leaf extract-derived ZnO nanoparticles synthesized from fresh and dried *R. acetosa* were evaluated against five different bacterial strains using the microdilution method [Supplementary Figures S1 and S2]. The MIC values for fresh leaf extract-derived ZnO nanoparticles from *R. acetosa* ranged from 14.20 to 113.64 µg/mL, while those for samples prepared from dried leaf extract-derived ZnO nanoparticles from *R. acetosa* ranged from 7.10 to 113.64 µg/mL [Table 4]. For fresh leaf extract-derived ZnO nanoparticles, the lowest reported MIC was 14.20 µg/mL, observed against both *E. faecalis* and *P. aeruginosa*. The MIC values were 56.82 µg/mL against both *S. aureus* and *K. pneumoniae*, and 113.64 µg/mL against *E. coli* (Table 4). 

Dried leaf extract-derived ZnO nanoparticles from *R. acetosa* showed the lowest MIC (7.10 µg/mL) against *E. faecalis*. For dried leaf extract-derived ZnO nanoparticles, the MIC was 28.41 µg/mL for *P. aeruginosa*, 56.82 µg/mL for *K. pneumoniae*, and 113.64 µg/mL for *S. aureus* and *E. coli* [Table 4]. Both preparations had identical reported MIC values against *E. coli* and *K. pneumoniae* (Table 4). ZnO nanoparticles synthesized using fresh *R. acetosa* leaf extract showed MIC values one twofold dilution step lower than those synthesized using dried leaf extract against *S. aureus* and *P. aeruginosa*. In contrast, the dried leaf extract-derived preparation showed an MIC one twofold dilution step lower against *E. faecalis*. Comparative findings should be interpreted cautiously given the limited replication and experimental conditions.

## 4. Discussion

The antibacterial activity of ZnO nanoparticles is determined by interrelated physicochemical properties such as particle size, surface area, crystal structure, surface defects, Zn^2+^ ion release, and reactive oxygen species (ROS) formation [39,40]. Recently, in green synthesis studies by different researchers, the composition of the extract has been reported to be responsible for these properties [5,6]. Abdelmigid et al. also reported variation in antibacterial activity across bacterial strains, with chemically synthesized ZnO nanoparticles producing slightly larger inhibition zones against MRSA than against the Gram-negative strains tested. For nanoparticles synthesized using pomegranate peel and coffee ground extracts, FTIR findings suggested that protein-associated hydroxyl and carboxyl groups could contribute to nanoparticle formation and stabilization [41]. Similarly, Şahin et al. reported a correlation between the physical properties of ZnO nanoparticles synthesized with Thymus syriacus [42] extract and their antibacterial activity against Gram-positive and -negative bacteria. Previous studies have suggested that smaller nanoparticles may interact more easily with bacterial surfaces and contribute to oxidative damage. Larger specific surface areas may facilitate contact and interaction with bacterial cell surfaces [43,44]. Likewise, researchers reported that surface defects in Zn nanoparticles can disrupt cell membrane integrity by promoting ROS production, and these nanoparticles can damage the bacterial membrane during membrane translocation [45,46]. Consistent with this finding, Naqvi et al. reported that oxygen vacancies in ZnO nanoparticles promote ROS production, and this process, along with the release of Zn^2+^ ions, may cause damage to the *S. aureus* cell membrane [47]. Dönmez et al. reported that ZnO nanoparticles synthesized with grape seed extract rich in phenolic compounds showed antioxidant and antibacterial activity against *S. aureus* [48]. Similarly, ZnO nanoparticles synthesized using pomegranate peel exhibit antimicrobial activity, supporting the belief that plant-derived compounds may contribute to nanoparticles’ biological activity [7]. In the present study, ZnO nanoparticles synthesized using fresh *R. acetosa* leaf extract showed a slightly lower MIC against *S. aureus* than those synthesized using dried leaf extract; this difference was limited to one twofold dilution step. 

On the other hand, Leta et al. reported that ZnO nanoparticles synthesized from different plant sources showed the highest activity against *E. faecalis* among the tested bacteria, and that particle size, surface chemistry, and extract-derived coating compounds may have contributed to this selective activity [49]; however, the lower MIC of ZnO nanoparticles from dried *R. acetosa* against *E. faecalis* suggests that antibacterial activity does not depend solely on particle size. Furthermore, in their study comparing ZnO nanoparticles of different sizes, Mirhosseini et al. detected one of the strongest antimicrobial effects on *E. faecalis* and reported that even relatively large nanoparticles could produce significant inhibition of this bacterium [50]. Similarly, Djearamane et al. showed that ZnO nanoparticles accumulated more on the cell surface of *E. faecalis* and led to greater growth inhibition than in Gram-negative *S. marcescens* [51], a sensitivity that they attributed to the nanoparticles’ adherence to the cell surface, interaction with functional groups in the cell wall, and disruption of membrane integrity. In the present study, the ZnO nanoparticles synthesized using dried *R. acetosa* leaf extract showed a lower MIC against *E. faecalis* despite their larger particle size; also, in our study, ZnO nanoparticles synthesized with fresh and dried leaf extract-derived *R. acetosa* exhibited lower MIC values (14.20, 28.41 µg/mL, respectively) against the *P. aeruginosa* strain. These variations were limited to one twofold dilution step. Previous studies report that green-synthesized ZnO nanoparticles show antibacterial activity against clinical isolates of *P. aeruginosa* and that nanoparticle application disrupts membrane integrity by increasing bacterial inner- and outer-membrane permeability [52]. Although the lipopolysaccharide-containing outer membrane of Gram-negative bacteria may limit nanoparticle entry into the cell, interactions between ZnO nanoparticles and the bacterial surface may contribute to antibacterial activity against *P. aeruginosa*. Abdelmigid et al. found that ZnO nanoparticles synthesized with pomegranate peel and coffee ground extracts have antibacterial activity against *P. aeruginosa* [41]. Lee et al. also reported that ZnO nanoparticles suppressed pyocyanin production and quorum sensing-related processes in P. aeruginosa via Zn^2+^ ions, while Mendes et al. suggested that electrostatic interactions between ZnO nanoparticles and the bacterial surface may contribute to membrane damage and disruption of cellular function, along with ROS production and Zn^2+^ release [40,53]. In this study, ZnO nanoparticles synthesized using fresh and dried leaf extract-derived R. acetosa showed similar antibacterial profiles against E. coli and K. pneumoniae, with MIC values of 113.64 µg/mL and 56.82 µg/mL, respectively. Ahmad et al. reported concentration-dependent antibacterial activity of ZnO nanoparticles against both *E. coli* and *K. pneumoniae* [54]. Furthermore, Abdelmigid et al. reported that ZnO nanoparticles synthesized using pomegranate peel and coffee ground extracts showed antibacterial activity against *K. pneumoniae* [41]. In a similar study, Hameed et al. demonstrated that ZnO nanoparticles suppressed bacterial growth by causing disorder and increased membrane permeability in the *E. coli* cell membrane [55]. In addition, Rasha et al. reported that ZnO nanoparticles synthesized using *Aspergillus niger* showed antibacterial activity against carbapenem-resistant *K. pneumoniae*, caused morphological changes in bacterial cells, and promoted wound healing in infected rats [56]; similarly, Reddy et al. reported that ZnO nanoparticles suppressed *K. pneumoniae* growth [57]. Plant-derived materials being investigated as sources for ZnO nanoparticle synthesis, including plant waste, may offer a more environmentally friendly approach by reducing reliance on hazardous chemical additives. Mathizhagan et al. reported that ZnO nanoparticles synthesized from tea waste exhibited antimicrobial activity, showing that plant waste can be used to produce functional nanomaterials [58]. Similarly, ZnO nanoparticles synthesized using *Euphorbia hirta* leaves, *Clitoria ternatea* flowers, *Solanum lycopersicum* fruits, *Brassica oleracea* var. botrytis leaves and *Pisonia alba* leaves have been reported to exhibit antibacterial activity [9,59,60,61,62]. These findings support using different plant materials for green synthesis of ZnO nanoparticles. 

Sowbarnika et al. reported that fresh or dried leaf extracts influenced the properties of biosynthesized ZnO nanoparticles, including their surface characteristics [13]. EDX analysis revealed that Zn and O were dominant in the sample synthesized from dried plant material, while the carbon content was higher in that from fresh material. The high carbon content detected in the fresh sample suggests that the organic components in the plant extract may be more densely retained on the nanoparticle surface, a finding consistent with the FTIR results, which showed more distinct bands in the 3380, 1580, 1415, and 1030 cm^−1^ regions in the fresh sample. The Zn–O vibrational band determined at approximately 435 cm^−1^ in both samples confirms the formation of ZnO nanoparticles. 

The experimental data indicated that ZnO NPs synthesized from fresh and dried leaf extracts differ in particle size (FE-SEM), crystallite size and crystallinity (XRD), and surface organic matter content (EDX, FT-IR). Both samples showed characteristic ZnO UV absorption and optical band gap values. Both materials inhibited all tested strains, and their MICs were identical for *E. coli* and *K. pneumoniae* and differed by only one twofold dilution for *S. aureus*, *E. faecalis,* and *P. aeruginosa*. Given the limited replication, the reproducibility and biological relevance of these MIC differences remain to be established. The following possible explanations for the strain-dependent tendencies should therefore be regarded as hypotheses that were not directly tested here: (i) the smaller primary particles and lower crystallinity of the fresh leaf extract-derived ZnO NPs may provide a larger reactive surface and more surface defects, which favor reactive oxygen species generation and Zn^2+^ release; (ii) the organic layer retained on the fresh leaf extract-derived ZnO NPs, which is also reflected in its more negative zeta potential (≈−24 mV vs. ≈−14 mV for dried leaf extract-derived ZnO NPs), may modify particle–cell interactions, surface charge and dissolution behavior; and (iii) residual extract-derived compounds may contribute directly to the activity. 

## 5. Conclusions

In this study, ZnO nanoparticles were successfully synthesized from fresh and dried leaf extract-derived *R. acetosa*, and their physicochemical and antibacterial properties were characterized. The dried extract-derived NPs were considerably more crystalline (crystallite size: 16.7 nm) than those from the fresh extract (3.0 nm), which showed markedly greater peak broadening, dislocation density, and microstrain. Both samples showed characteristic ZnO UV absorption and optical band gaps of 3.15 and 3.17 eV. EDX and FTIR analyses revealed that ZnO NPs synthesized using fresh leaf extract-derived R. acetosa contained a substantial organic fraction (52.71 at.% C), whereas the dried extract NPs were Zn-rich. FE-SEM images and ImageJ analysis showed particle sizes of 21.6 ± 6.1 and 14.9 ± 3.6 nm for the dried and fresh leaf extract NPs, respectively. DLS revealed hydrodynamic sizes of approximately 69 and 51 nm and zeta potentials of approximately -14 and −24 mV for the dried and fresh leaf extract NPs, respectively. The findings reveal that ZnO nanoparticles synthesized using fresh and dried leaf extract-derived *R. acetosa* exhibit antibacterial activity against both Gram-positive and Gram-negative bacteria. Based on their structural and optical properties, dried leaf extract-derived ZnO NPs may be suitable for further evaluation in photocatalytic, UV-protection, and sensing applications, while the antibacterial activity of fresh leaf extract-derived ZnO NPs suggests potential for antibacterial coatings, wound dressings, and active food packaging. However, these applications were not evaluated in the present study and require further experimental investigation.

## Figures and Tables

**Table 1:** EDX atomic (%) results of the ZnO NPs synthesized with dried and fresh *Rumex* spp. leaf extracts.

Element	Atomic (%) Dried Leaf Extract-Derived ZnO NPs	Atomic (%) Fresh Leaf Extract-Derived ZnO NPs
C	-	52.71
O	44.32	35.84
Zn	55.68	11.45

**Table 2:** The crystallite size (D), dislocation density (δ), and strain value (ε) of the ZnO NPs synthesized with dried and fresh *Rumex* spp. leaf extracts.

Sample	2-θ (°)	FWHM (°)	D (nm)	δ (10^16^ Lines/m^2^)	ε (10^−3^)
ZnO NPs (dried *Rumex* spp. leaf)	36.24	0.50	16.69	0.36	2.08
ZnO NPs (fresh *Rumex* spp. leaf)	36.48	2.79	3.00	11.10	11.56

**Table 3:** The lattice parameters and crystallite sizes of the ZnO NPs synthesized with dried and fresh *Rumex* spp. leaf extracts.

	a = b (Å)	c (Å)	Unit Cell Volume (Å^3^)	Crystallite Size (nm)
ZnO NPs (dried *Rumex* spp. leaf)	3.251	5.211	47.69	17.5
ZnO NPs (fresh *Rumex* spp. leaf)	3.272	5.217	48.38	1.60

**Table 4:** Antibacterial activity of fresh and dried leaf extract-derived *Rumex acetosa* Against ATCC Reference Strains.

Synthesized Sample (µg/mL)	ATCC Reference Strains (µg/mL)				
	*S. aureus*	*E. faecalis*	*E. coli*	*K. pneumoniae*	*P. aeruginosa*
Fresh leaf extract-derived ZnO nanoparticles	56.82	14.20	113.64	56.82	14.20
Dried leaf extract-derived ZnO nanoparticles	113.64	7.10	113.64	56.82	28.41

## Data Availability

The data supporting the findings of this study are included in the article.

## Supplementary Materials

The following supporting information can be downloaded at https://www.mdpi.com/article/10.3390/nano16211441/s1: Figure S1. Antibacterial activity of ZnO nanoparticles synthesized using dried and fresh leaf extracts against reference bacterial species. Figure S2. Antibacterial activity of ZnO nanoparticles synthesized using fresh leaf extract against reference bacterial species.

## Abbreviations

The following abbreviations are used in this manuscript:

ZnO NPs	Zinc oxide nanoparticles
UV–Vis	Ultraviolet–visible spectroscopy
FE-SEM	Field emission scanning electron microscopy
EDX	Energy-dispersive X-ray spectroscopy
XRD	X-ray diffraction
