# Supplementary material for: Green Synthesis of ZnO Nanoparticles Using Fresh and Dried Rumex acetosa Leaf Extracts: Characterization and Antibacterial Activity
Source: Nanomaterials (Basel). 2026 Nov 6;16(21):1441. doi: 10.3390/nano16211441 (PMC13765186; doi:10.3390/nano16211441)
Supplement: Supplementary file 1 [file nanomaterials-16-01441-s001.zip › nanomaterials-4631583-supplementary.pdf]

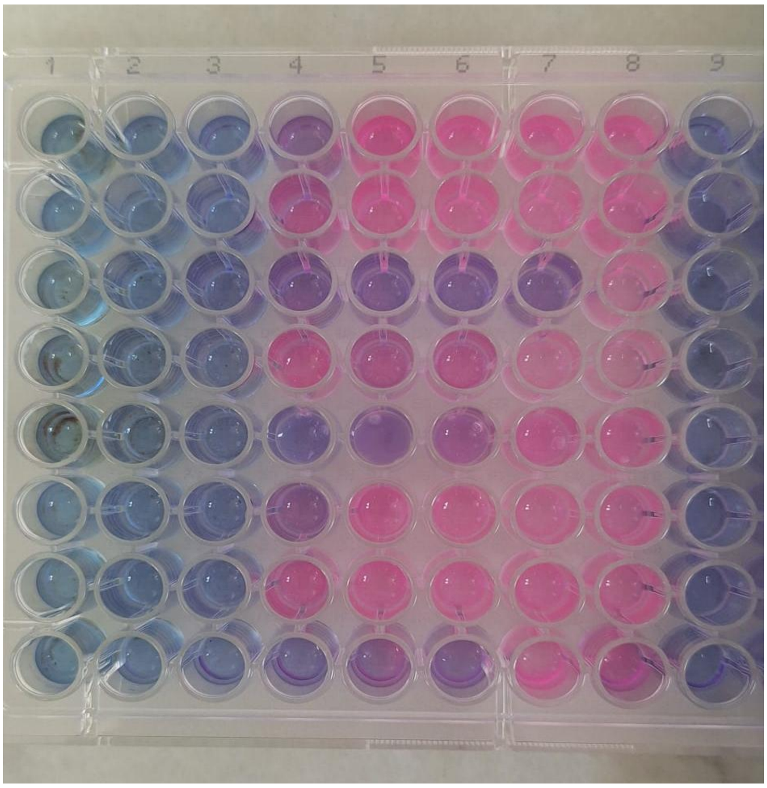

Figure S1. Antibacterial activity of ZnO nanoparticles synthesized using dried and fresh leaf extracts against reference bacterial species. (The assay was performed left to right, from 1 to 8. Dried samples (A: K. pneumoniae (ATCC 27736), B: E. coli (ATCC 25922), C: E. faecalis (29212), D: S. aureus (ATCC 29213), E: P. aeruginosa (ATCC 27853). Fresh samples (F: K. pneumoniae (ATCC 27736), G: E. coli (ATCC 25922), H: E. faecalis (29212). Column 9: Sample control: Nanoparticle, Resazurin, and MHB.)

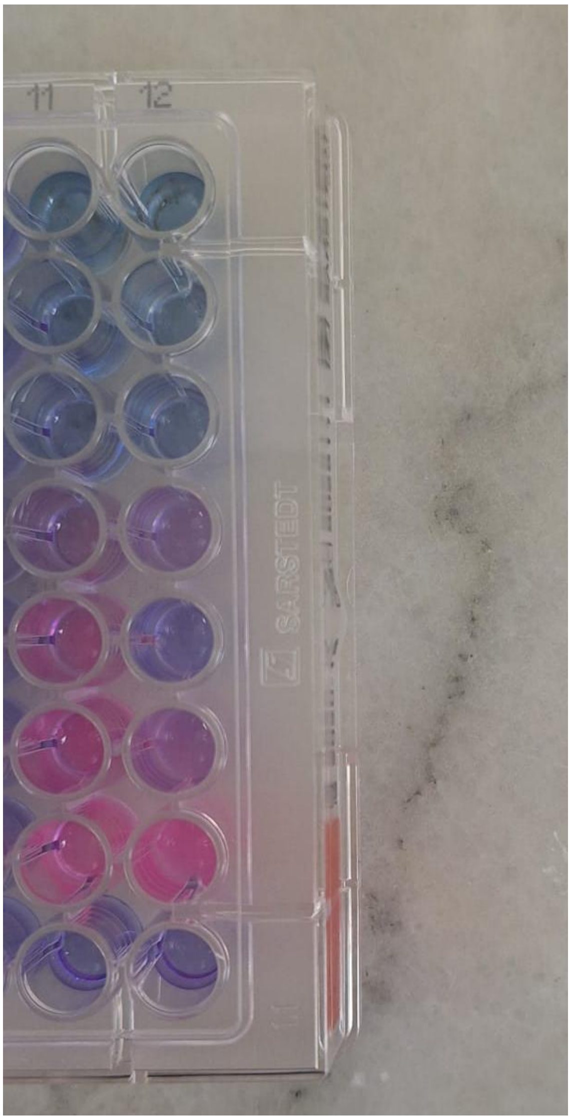

Figure S2. Antibacterial activity of ZnO nanoparticles synthesized using fresh leaf extract against reference bacterial species. (Column 11: S. aureus (ATCC 29213), Column 12: P. aeruginosa (ATCC 27853). The last wells in both rows serve as controls: nanoparticles, resazurin, and MHB. The assay was performed top to bottom.)
